# The Origins of Specificity in Polyketide Synthase Protein Interactions

**DOI:** 10.1371/journal.pcbi.0030186

**Published:** 2007-09-28

**Authors:** Mukund Thattai, Yoram Burak, Boris I Shraiman

**Affiliations:** 1 National Centre for Biological Sciences, Bangalore, India; 2 Kavli Institute for Theoretical Physics, University of California Santa Barbara, Santa Barbara, California, United States of America; Stanford University, United States of America

## Abstract

Polyketides, a diverse group of heteropolymers with antibiotic and antitumor properties, are assembled in bacteria by multiprotein chains of modular polyketide synthase (PKS) proteins. Specific protein–protein interactions determine the order of proteins within a multiprotein chain, and thereby the order in which chemically distinct monomers are added to the growing polyketide product. Here we investigate the evolutionary and molecular origins of protein interaction specificity. We focus on the short, conserved N- and C-terminal docking domains that mediate interactions between modular PKS proteins. Our computational analysis, which combines protein sequence data with experimental protein interaction data, reveals a hierarchical interaction specificity code. PKS docking domains are descended from a single ancestral interacting pair, but have split into three phylogenetic classes that are mutually noninteracting. Specificity *within* one such compatibility class is determined by a few key residues, which can be used to define compatibility subclasses. We identify these residues using a novel, highly sensitive co-evolution detection algorithm called CRoSS (correlated residues of statistical significance). The residue pairs selected by CRoSS are involved in direct physical interactions in a docked-domain NMR structure. A single PKS system can use docking domain pairs from multiple classes, as well as domain pairs from multiple subclasses of any given class. The termini of individual proteins are frequently shuffled, but docking domain pairs straddling two interacting proteins are linked as an evolutionary module. The hierarchical and modular organization of the specificity code is intimately related to the processes by which bacteria generate new PKS pathways.

## Author Summary

Biomolecular interactions can be extraordinarily specific. In many instances, a protein can select its single correct binding partner from among a large array of closely related candidates. For polyketide synthases (PKSs), a family of bacterial enzymes, such specificity is essential. Like workers on an assembly line, PKSs function as multiprotein chains, each enzyme modifying its substrate before passing it along to the next. And like a well-designed jigsaw puzzle, the overall multiprotein chain is correctly ordered precisely because each component protein can only bind to specific nearest neighbors. A PKS multiprotein chain is held together by sticky “head” and “tail” domains found at either end of each protein, the head of one protein binding to the tail of the next. We looked for patterns in the amino-acid sequences of these domains that could explain why certain head–tail pairs bind, while others do not. We discovered that heads and tails each come in three very different varieties. Mismatched head–tail pairs do not bind at all, while the binding of a matching head–tail pair is governed by the amino acids found at a few key positions on the physical interface between these domains.

## Introduction

The extraordinary biosynthetic capabilities of polyketide synthases (PKS), some of the largest known bacterial multi-enzyme complexes, have been extensively investigated over the past decade. Using a combination of biochemical and genetic techniques, researchers have developed a detailed understanding of the organization and structure of these complex enzymes, as well as of the biochemical reactions they catalyze [1]. The assembly of a polyketide proceeds by the successive addition of acyl extender groups to a growing biochemical polymer. Each module of a modular PKS is a multidomain catalytic unit responsible for a single step of polyketide chain extension. PKS proteins can each contain one or more modules. Different modules add different basic or modified extender units, so the order of proteins and their modules in the multiprotein chain determines the chemical structure of the final polyketide product. Crucially, PKS catalytic domains exhibit broad substrate tolerance, and are able to extend “unnatural” polyketide substrates [2,3]. In particular, PKS modules are catalytically active even when their order is changed, enabling a combinatorial diversity of possible polyketide products. This property, which suggests that modules are frequently shuffled during natural selection, has generated enormous interest in using PKS pathways to achieve combinatorial biochemistry in the laboratory [2–6].

Biochemical studies of PKS systems have recently been complemented by powerful computational tools. These tools allow results from well-characterized PKS systems to be extended to the rapidly growing set of putative PKS gene clusters in fully sequenced bacterial genomes. Computational analysis of sequence data has been used to identify catalytic domains and to predict their substrate specificity, both for PKS systems [7,8] as well as for the closely related nonribosomal peptide synthase (NRPS) systems [8,9]. In a recent analysis, Minowa et al. [10] were able to effectively predict the catalytic function of individual proteins, as well as the order in which multiple proteins act to produce the final polyketide product, by combining a variety of data including the chromosomal context of genes, and the sequences and phylogeny of catalytic and linker regions. While these predictive tools can be extremely accurate, their output cannot necessarily be interpreted in ways that provide biological insight. It is therefore important, in parallel with predictive approaches, to investigate underlying evolutionary and molecular mechanisms. For example, a new generation of sequence-based classification algorithms promises not only to have predictive power, but also to reveal sequence features that are important for discrimination, thus providing insight at the molecular level [11]. Comparative sequence analysis can also be used to shed light on the evolutionary history of a system, as shown in a recent study of PKS catalytic-domain duplication [12].

Here we use sequence data to investigate PKS protein interactions. For the PKS proteins to line up in the correct order, protein interactions must be *specific* [13,14]: certain protein pairs must be allowed to bind, while others must be prevented from doing so. It is remarkable that these proteins are able to correctly discriminate between various possible binding partners, given that all the docking domains which mediate their interactions are homologous. We uncover key sequence features that govern PKS protein interactions, and thereby construct a predictive specificity code. We show that, by studying how the code is organized, we can learn a great deal about the evolutionary origins as well as the molecular basis of this elegant specificity.

## Results

### Docking Domains and Interaction Compatibility Classes

Biochemical investigations have shown that interactions between PKS proteins map to docking domains at their N- and C-termini [13–15]. Starting with a seed alignment of terminal fragments from well-characterized PKS pathways [7], we used PSI-BLAST to assemble a dataset of proteins with regions homologous to these (Text S1, Section 1). Homologous regions were only detected at protein termini, never in the interior. Most of the proteins we pulled up belonged to biochemically characterized modular PKS pathways, and the rest to putative modular PKS pathways in fully sequenced genomes. Almost all known modular PKS proteins contained homologous termini, and we found no significant hits to non-PKS proteins. This suggests that all modular PKS proteins employ the same mechanism to mediate interactions.

**Figure 1:**
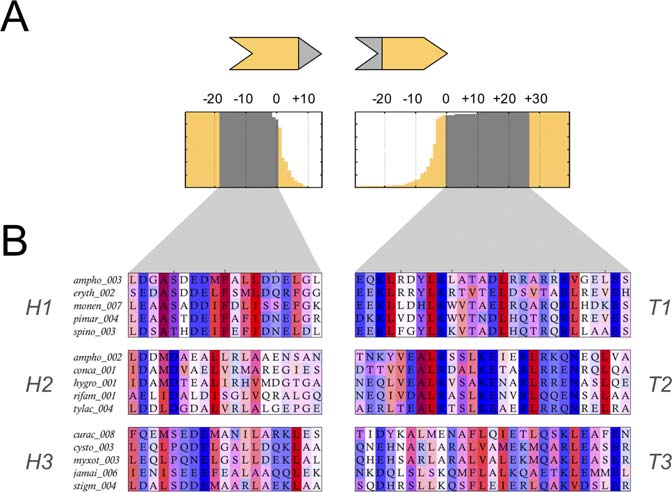
PKS Docking Domains (A) Location of docking domains at protein termini. A pair of interacting PKS proteins are represented as chevrons, with their docking domains indicated in grey: the C-terminal head domain (pointed) and the N-terminal tail domain (notched). The boxes immediately below give an overview of the 149 head and tail domains in our dataset. Each horizontal line represents a single protein sequence; the sequences are aligned according to their conserved docking domains (grey), and sorted according to the lengths of their unconserved overhangs. We see that most docking domains are to be found within a few amino acids of protein termini. (B) Representative multiple-sequence alignments of the 19 aa C-terminal head domains (left) and 27 aa N-terminal tail domains (right). Hydrophobic residues are colored red, hydrophilic residues are colored blue, and intensity reflects sequence conservation [29]. Note that sequences are sorted differently than in Figure 1A. Each row shows an interacting head–tail pair, labeled by the PKS pathway it belongs to, and the interface number within that pathway. For example, *ampho_003* represents the interface between proteins 3 and 4 of the amphotericin PKS. (Pathway abbreviations are described in detail in Dataset S1.) The head and tail alignments are divided into three groups each (*H1*, *H2*, *H3,* and *T1*, *T2*, *T3*) corresponding to the clusters discussed in Figure 2.

**Figure 2:**
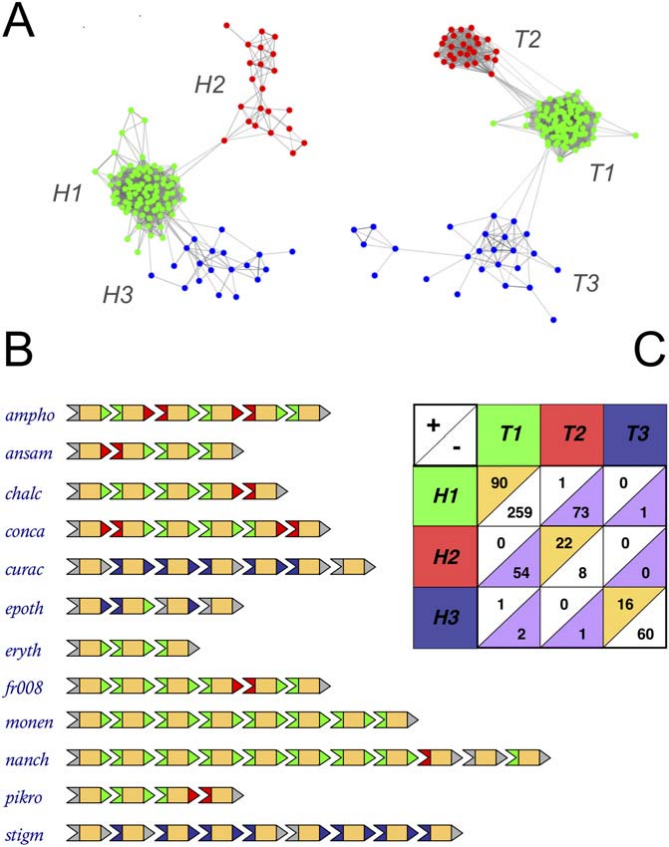
Docking Domain Compatibility Classes (A) Docking domains are clustered according to sequence similarity (Text S1, Section 2). Each node represents a particular head (left) or tail (right) domain; two domains are connected by a line if their BLAST e-value is less than a defined cutoff (2.0e-4 for heads, and 1.0e-4 for tails). Head and tail domains each independently assort into three phylogenetic clusters, labeled *H1*, *H2*, *H3*, and *T1*, *T2*, *T3*, respectively. For the moment, cluster coloring is arbitrary. (B) Examples of PKS multiprotein chains. Each row shows a different PKS pathway, with names as defined in Dataset S1. Proteins are represented as chevrons, with C-terminal head domains (pointed) and N-terminal tail domains (notched) now colored according to their phylogenetic group, as defined in Figure 2A. The pathway termini, as well as domains which could not be clustered, are colored grey. Note that interactions predominantly occur between docking domains of the same color. There are only two exceptions to this rule, one of which is shown in the *nanch* multiprotein chain. (The other, in the *nidda* multiprotein chain, involves a domain that lies at the boundary of its parent cluster, indicating that it has probably been misclassified by our clustering algorithm; see Dataset S2.) Actinobacterial pathways tend to use domains pairs of type *H1*–*T1* and *H2*–*T2*, while myxobacterial and cyanobacterial pathways use domain pairs of type *H3*–*T3* alone. (Again, the few exceptions to this rule, such as in the *epoth* multiprotein chain, are likely due to misclassification.) (C) Phylogenetic clusters coincide with docking domain compatibility classes. Each PKS pathway in our dataset gives us a list of known interactors, as well as a list of known noninteractors. For example, the amphotericin pathway contains five internal head and tail domains; of the 25 possible pairings of these domains, five represent interactors (three *H1*–*T1* and two *H2*–*T2*), while the remaining 20 represent noninteractors (six *H1*–*T1*, six *H1*–*T2*, six *H2*–*T1*, and two *H2*–*T2*). We tallied such interaction and noninteraction information over the 42 PKS pathways in our dataset, and summarized our results in a single table. Each row corresponds to a head cluster, and each column to a tail cluster. The top-left entry of every cell reports the number head–tail pairs of the given variety known to be interactors; the bottom-right entry reports the number of head–tail pairs known to be noninteractors. For example, we know one interactor and 73 non-interactors of the *H1*-*T2* variety. The correspondence between the head and tail clusters is obvious: we find large numbers of interactors within compatible clusters (on-diagonal, highlighted in orange) and large numbers of noninteractors between them (off-diagonal, highlighted in purple). This defines a one-to-one pairing of head and tail clusters into three compatibility classes, *H1*–*T1* (green), *H2*–*T2* (red), and *H3*–*T3* (blue), and justifies the common coloring used in Figure 2A. This division into compatibility classes is a useful predictive tool for actinobacterial pathways, since they tend to contain docking domains of multiple varieties. For example, since the *ampho* pathway has three *H1*–*T1* domain pairs and 2 *H2*–*T2* domain pairs, only 12 of the 120 possible ways of pairing them are compatible (2!3! out of 5!). Of the 33 actinobacterial pathways in our dataset, 19 contain both *H1*–*T1* and *H2*–*T2* varieties (Dataset S2). For these mixed pathways, on average less than a third of all possible ways of pairing their domains are compatible.

We next confined our attention to 42 biochemically characterized modular PKS pathways in which the order of proteins within the multiprotein chain has been determined (Dataset S1). An alignment of the protein termini revealed short, conserved regions at the very ends of the proteins (Figure 1A): a 19 aa C-terminal “head” region, and a 27 aa N-terminal “tail” region (Figure 1B). Throughout our discussion, we shall refer to these regions as docking domains. (The docking domains originally defined by Broadhurst et al. [15] are slightly larger protein interaction regions, of which the heads and tails defined here form only a part: PKS protein termini typically contain one conserved N-terminal helix, which corresponds to our tail domain, and three conserved C-terminal helices, of which the most C-terminal helix corresponds to our head domain.) When these domains were clustered according to sequence similarity [16] (Text S1, Section 2), the heads and tails each *independently* assorted into three phylogenetic groups (Figure 2A). When the docking domains of the proteins in each multiprotein chain were labeled according to group membership (Figure 2B), a striking pattern emerged: phylogenetic clustering coincided precisely with head–tail interactions (Figure 2C). Heads from one group could only interact with tails from a corresponding group, but were incompatible with other tails, and vice versa. From this one-to-one pairing, we were able to assign common labels to the head and tail clusters, thus defining three mutually incompatible classes of docking domain pairs: *H1*–*T1*, *H2*–*T2*, and *H3*–*T3*. However, membership within a compatibility class was only a necessary, not a sufficient, condition for head–tail interaction: there were still numerous cases in which domain pairs belonging to the same compatibility class did not interact (Figures 2B and 2C). This implies that, *within* each class, there are additional rules that determine the set of allowed interactions. In essence, there is another layer to the PKS specificity code.

### CRoSS: Sensitive Detection of Co-Evolving Residues

To gain further insight into the rules that governed specificity, we sought to identify residue pairs that co-evolved between interacting partners [17,18]. This task is complicated by the fact that our dataset is small and nonuniformly sampled, therefore dominated by spurious correlations. To overcome this problem, we developed a new algorithm called CRoSS (correlated residues of statistical significance) which uses both interaction and noninteraction data to identify significant pairings between head and tail residues (Methods). For each site pair, the algorithm first calculates, separately for interactors and noninteractors, the joint distribution of amino acids summed over pathways. It then assigns a score to that site pair, which is essentially a *p*-value reporting the significance of the difference between these distributions. CRoSS has numerous advantages over existing co-evolution algorithms [17,18] (Figure S1D). Because it is based on comparisons within rather than between pathways, it is less susceptible to errors from nonuniform sampling; because it reports a significance rather than a correlation, it can be applied to datasets of any size; and because it identifies sites but averages over amino acids, it is more sensitive given smaller datasets. CRoSS can generally be used to investigate protein specificity whenever data are available about which protein pairs do or do not interact, such as for bacterial two-component systems [19].

### Code Words and Interaction Compatibility Subclasses

**Figure 3:**
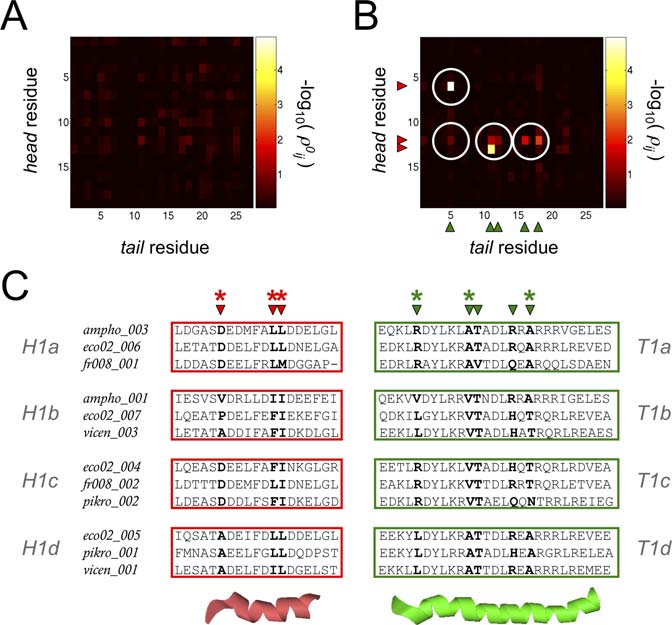
Co-Evolving Residues In this figure, symbols associated with head domains are colored red, and those associated with tail domains are colored green. This coloring is unrelated to compatibility class, as all domains pictured belong to compatibility class *H1*–*T1*. (A,B) CRoSS matrices, showing the residue pairs that significantly contribute to specificity (Methods). Residues on the C-terminal head domain are indexed vertically (*i* = 1, ... , 19); residues on the N-terminal tail domain are indexed horizontally (*j* = 1, ... , 27). Each entry shows -log_10_(*ρ_ij_*) for the corresponding site-pair, with the scale indicated on the color bar; the higher this value, the more significant the site-pair as a determinant of specificity. (A) The *H1*–*T1* control matrix, generated by comparing random pairings with noninteractors. Since we expect no significant hits, these entries provide us with an estimate of the random background. (B) The *H1*–*T1* interaction matrix, generated by comparing interactors with noninteractors. Several entries are highlighted above the background (white circles serve as guides to the eye). The matrix is sparse, showing that residue pairs at a few key sites vary independently, uncorrelated to any broad phylogenetic patterns. There are only seven significant residue pairs (Figure S1C). These are, in order of significance, {*i*, *j*} = {6,5}, {13,11}, {12,18}, {12,11}, {12,12}, {12,16}, {12,5}. The three head residues and five tail residues that make up these pairs are indicated along the axes by red and green arrows, respectively. (C) Representative multiple-sequence alignments of *H1* head domains (left) and *T1* tail domains (right), with cartoon representations of the head and tail domains shown below. Each row shows an interacting head–tail pair, labeled by the PKS pathway it belongs to, and the interface number within that pathway. The three head residues and five tail residues selected by CRoSS are indicated by red and green arrows, respectively; the corresponding positions in the sequence alignments are highlighted in bold. The three most significant head and tail residues (used to define code words in Figure 4) are indicated by asterisks. The head and tail alignments are divided into four groups each (*H1a*–*H1d* and *T1a*–*T1d*) corresponding to the subclasses discussed in Figure 4.

We used CRoSS to investigate the key residues that determine specificity within each compatibility class. We were able to detect significant residue pairs only for class *H1–T1* (Figures 3A and 3B). (This does not mean there are no correlations in the other cases, only that we cannot detect them with confidence given the smaller sizes of those datasets.) We found that the *H1–T1* correlation matrix was extremely sparse (Figure 3B), showing that a small number of site pairs co-evolved independently, uncorrelated to any broad phylogenetic patterns. CRoSS identified only seven significant correlated residue pairs for *H1–T1* interactors, involving three head residues and five tail residues (Figure S1C). Note that while residues involved in protein secondary structure are expected to be highly conserved, those that determine specificity should show moderate sequence conservation but strong co-evolution. Such residues cannot be identified from structure alone, but can only be identified from the type of correlation analysis presented here, or from detailed domain swapping and mutagenesis experiments [20,21].

**Figure 4:**
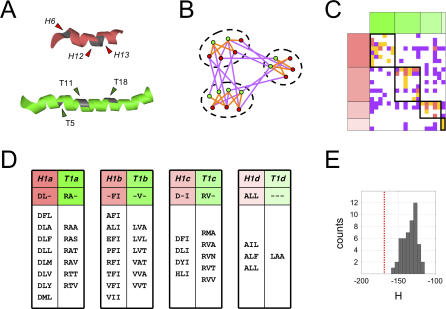
Code Words and Compatibility Subclasses In this figure, symbols associated with head domains are colored red, and those associated with tail domains are colored green. This coloring is unrelated to compatibility class, as all domains pictured belong to compatibility class *H1*–*T1*. (A) The three most significant CRoSS pairs pick out three residues each on the head (red) and tail (green) domains. These residues (indicated by asterisks in Figure 3C) are highlighted by arrows, with their position along the domain shown in parentheses. The amino acids at these positions define our code words. (B) Schematic representation of code word clusters. Each node of the graph represents a unique head (red) or tail (green) code word, and each edge represents a known interaction (orange) or noninteraction (purple) between code words. We use a Monte Carlo algorithm (Text S1, Section 3) to group the nodes into clusters, such that interactions are enriched within a cluster, and noninteractions are enriched between clusters. These clusters thus represent a refinement of *H1-T1* into compatibility subclasses. (C) Actual code word clusters. This is a matrix representation of the interaction graph shown in Figure 4B. Each row corresponds to a head code word (red), and each column to a tail code word (green). The entries represent edges, showing that the corresponding code word pairs have been found on known interactors (orange), known noninteractors (purple), both (pink), or neither (white). Code words are grouped into four clusters (each labeled by a different shade of red or green), corresponding to subclasses of *H1*–*T1* within which interactions are enriched. Nodes that occur as singletons are not shown. (D) Synonymous sets of code words. The code words belonging to each subclass are explicitly listed, in the same order as in the matrix of Figure 4C. Comparison with the matrix shows that, within a given subclass, each head is compatible with several tails, and vice-versa. The subclasses are labeled by shade, as well as by the index *a*, *b*, *c*, *d*. Within each subclass, we see a high degree of code word sequence similarity. If an amino acid occurs in a majority of instances at a given position, it is included in the consensus sequence characterizing a given subclass. (E) Histogram of clustering energies for 50 datasets with randomized interactions (Text S1, Section 3). The more negative the energy, the better the clustering. The red line indicates the energy of the true dataset, far to the left of the distribution for randomized datasets. This indicates that the observed degree of clustering is statistically significant, with *p*-value < 0.02.

Having identified the sites predominantly responsible for *H1–T1* specificity, we next asked whether we could construct a specificity code for interactions within this class. We focussed on the three most significant CRoSS pairs, which involved three residues each on the head and tail domains (indicated by asterisks in Figure 3C, and by arrows in Figure 4A). For any given head or tail, these residues define a short amino acid code word. We used a Monte Carlo technique to cluster these words into cliques [22], such that interactions were enriched within cliques but suppressed between them (Figures 4B and 4C; Text S1, Section 3). The code words broke up into clearly distinguishable sets of synonyms (Figure 4D), with sequences much more similar than expected by chance (*p*-value < 0.02, estimated by using datasets with randomly permuted interactions; Figure 4E). These clusters essentially correspond to a refinement of *H1–T1* into interaction compatibility subclasses (Figure 3C).

### Testing the Specificity Code

The organization of docking domains into compatibility classes and subclasses is striking, but perhaps circumstantial. Are there falsifiable predictions that can be tested against independent sources of data, other than those used during the original analysis? In this section, we present three tests of the specificity code. The first test is structural: by mapping our results onto an NMR structure of PKS docking domains, we ask whether the correlated residue pairs picked out by CRoSS involve actual physical interactions. The second test is statistical: we break up our data into training and test sets, using the former to make predictions, and the latter to validate them. The third test is functional: we use published experimental data involving hybrid PKS pathways to validate our classification of docking domains into compatibility classes.

#### Structural: Physically interacting residues.

**Figure 5:**
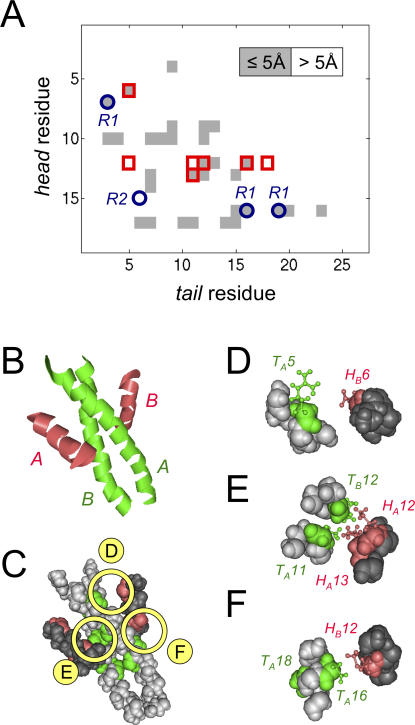
CRoSS Residues and Physical Interactions In (B–F), symbols associated with head domains are colored red, and those associated with tail domains are colored green. This coloring is unrelated to compatibility class, as all domains pictured belong to compatibility class *H1*–*T1*. (A) Comparison of the residues selected by CRoSS to those that are in physical contact in the docked domain NMR structure. The matrix has residues of the head domain indexed vertically, and residues of the tail domain indexed horizontally. Each entry is shaded gray if the pairwise distance between the corresponding residues is 5 Å or less, and white otherwise. The residue pairs selected by CRoSS are highlighted as red boxes; remarkably, four of the seven CRoSS pairs are separated by 5 Å or less. Residue pairs previously suggested as contributing to specificity are highlighted as blue circles: R1, suggested by Broadhurst et al. [15] and Weissman [20] as “code residue pairs” that play a critical role in discrimination; R2, demonstrated by Weissman [21] to alter the efficiency of docking in the erythromycin PKS. It has also been suggested that the entire complement of hydrophobic residues at the docking interface might contribute to specificity [20]. (B–C) Structure of the docking complex between the head domain of protein 2 (red) and the tail domain of protein 3 (green) of the erythromycin PKS [15]. PKS proteins exist as homodimers, so the docking complex involves a pair of tail domains *T_A_* and *T_B_* (which form a coiled coil) and a pair of head domains *H_A_* and *H_B_* (which are alpha helices that lock around the coiled coil). (B) Cartoon representation of the structure, showing domain labels. (C) Space-filling representation of the structure. The head domains are now colored dark grey, and the tail domains are colored light gray, while the head and tail residues selected by CRoSS are colored red and green, respectively. The three circles indicate regions that have been magnified in subsequent panels of the figure. (D–F) Space-filling representations of the structure, magnified around the neighborhood of the CRoSS residues. The side chains of the significant head (red) and tail (green) residues are also shown. As seen in Figure 5A, four out of the seven CRoSS residue pairs correspond to pairwise physical interactions: {*i*, *j*} = {6,5}, {12,12}, {13,11}, {12,16}. Note that the two copies of any residue participate in symmetric contacts, but it is not possible for CRoSS to assign which of two possible pairings, i.e., *A*–*A* and *B*–*B* versus *A*–*B* and *B*–*A*, actually occurs. For example, *H_B_*6 contacts *T_A_*5, while *H_A_*6 contacts *T_B_*5. One copy of each interacting pair is shown here: (D) *H_B_*6-*T_A_*5, (E) *H_A_*12-*T_B_*12, (E) *H_A_*13-*T_A_*11, (F) *H_B_*12-*T_A_*16. We have also highlighted (F) *T_A_*18, which does not lie on the head–tail interface but is instead buried within the tail coiled-coil. It is possible that destabilization of the *T_A_*18-*T_B_*18 contact changes the global conformation of the coiled-coil, and thus has a long-range effect on head–tail interactions. (Similar long-range effects have been demonstrated to alter the efficiency of docking in the erythromycin PKS [21].)

A key component of our analysis, information about which protein pairs in a PKS system do or do not interact, is based on PKS multiprotein chain order, which in turn is typically inferred from the nature of the polyketide synthesized by the system. Underlying this inference is the assumption that there is only one order in which the proteins can interact, namely that which results in the correct polyketide product. Alternatively, it could be argued that protein interactions are nonspecific, and that multiprotein chains of various permutations do arise, only one of which is catalytically active and therefore detectable. Under the former hypothesis of physical specificity, we might expect some of the co-evolving residue pairs detected by CRoSS to be involved in direct physical interactions. Under the latter hypothesis of chemical specificity, co-evolution would arise indirectly due to the constraints of catalysis, and would therefore have no relationship to physical interactions. In order to distinguish between these two possibilities, we mapped our *H1–T1* CRoSS residues onto the only available NMR structure of a PKS docking domain interaction complex [15], corresponding to an *H1–T1* interface between proteins 2 and 3 of the erythromycin synthase pathway (Text S1, Section 4). Remarkably, four of the seven CRoSS pairs mapped to residue pairs separated by 5 Å or less in the NMR structure (Figure 5). Of the 19 × 27 = 513 possible residue pairings between the head and tail domains, only 31 lie within this separation (Figure 5A). If we had selected seven residue pairs at random, we would have picked out four or more such proximate pairs with probability *p* = 3.4e-4. This highly significant agreement between sequence and structure provides strong, independent confirmation that CRoSS is picking out residue pairs involved in physical specificity. By the same token, it provides confidence that the structure inferred from NMR is an accurate description of the in vivo interaction complex.

#### Statistical: Training and test data.

Any successful predictive code must be able to generalize to previously unseen data. This ability can be measured by training the code on one subset of data (e.g., allowing it to learn sequence patterns), and testing its predictions against another (e.g., applying those pattern-based rules to predict interactions). The true positive rate (TP) is the fraction of known interactors predicted to interact, while the false positive rate (FP) is the fraction of known noninteractors predicted to interact. Random guessing would give TP = FP; a code which performed better than random would have TP > FP; the ideal code would have TP = 1, FP = 0. We carried out training and testing separately for the two levels of the specificity code (Text S1, Section 5). We found that predictions based on the classification of docking domains into compatibility classes gave TP = 0.97, FP = 0.52. That is, matching of compatibility class represents a necessary (almost all interacting pairs match) but not sufficient (half of the noninteracting pairs also match) condition for head*–*tail interaction, consistent with our earlier results. In contrast, predictions based on the division into subclasses performed only marginally better than random, with TP = 0.6, FP = 0.5 (Figure S2). When additional CRoSS residues were included in the construction of our code words, the performance was no better than random. Thus, while we were able to identify key residue *positions* with excellent statistical significance (as discussed in the previous section), the identification of predictive code words proved more difficult. The reason for this is that CRoSS averages over amino acids, while code word clusters are based on the particular amino acids found at each position. Since there are many distinct code words, the connections between them become sparse, leaving the algorithm unable to generalize from training data to test data. We do expect the predictive ability of the subclass code to improve once the size of the dataset is increased, and connections become better sampled.

#### Functional: Whole-protein hybrid PKS systems.

The hypothesis that docking domains from different compatibility classes cannot interact is certainly falsifiable: experimental evidence of a significant number of mismatched interactors would lead us to reject it. We see only two mismatched interactors in our dataset, one of which involves a domain that is probably misclassified (Figure 2C; Dataset S2). On the other hand, we have a large body of evidence in favor of it, including the statistical analysis presented above. To further test the validity of the hypothesis, we looked at cases in which hybrid systems were generated by combining whole proteins from different PKS pathways. There are several reported examples of functional interactions between hererologous proteins with compatible docking domains. These include *pikro_002*-*eryth_003* [23], *pikro_002*-*olean_003* [23], and *eryth_001*-*pikro_002* [24], all of which would require *H1–T1* docking (Figure 2B). To some extent, this is to be expected: successful hybrids tend to be made by combining closely related PKS sytems, so the sequential interactions of the parent multiprotein chains are recapitulated in the hybrid. More interestingly, an efficient nonsequential interaction was observed for *eryth_001*-*pikro_004* [24], a pairing that would seem to require *H1–T2* docking (Figure 2B), thus violating the compatibility class hypothesis. Closer inspection reveals that the situation is more complicated. A successful *eryth_001*-*pikro_004* interaction ought to result in polyketide chain elongation, similar to the natural interaction between *pikro_003* and *pikro_004*; instead, it results in polyketide chain cyclization and release, similar to the nonstandard interaction between *pikro_003* and a truncated form of *pikro_004* (which lacks its N-terminal docking domain) [25]. The fact that the N-terminal docking domain of *pikro_004* is unnecessary for the nonstandard *pikro_003*-*pikro_004* interaction suggests that it might be similarly unnecessary for the observed *eryth_001*-*pikro_004* interaction. However, this remains to be directly demonstrated. Since the nonstandard *pikro_003*-*pikro_004* interaction can occur even when the N-terminal docking domain of *pikro_004* is present [26], and since the C-terminal docking domain of *eryth_001* is necessary for the *eryth_001*-*pikro_004* interaction [24], the possibility of *H1–T2* docking cannot be ruled out. Nevertheless, when taken together, the results from hybrid pathways suggest that the classification of docking domains into compatibility classes is biologically meaningful.

## Discussion

We set out to understand the rules that governed interactions between modular PKS proteins. By focusing on the conserved N- and C-terminal docking domains, we were able to construct a predictive specificity code, and to prove that it pertained to pairwise physical interactions. This code provided us with necessary but not sufficient conditions for protein interaction, indicating that further determinants of specificity were required to explain the observed data. There have been several proposals regarding the nature of such determinants. First, it is possible that additional information is contained in the docking domains themselves (Figure 5A). For example, it has been suggested that interaction efficiency could be influenced by long-range electrostatic interactions between a set of “code residues” [15,20,21] and short-range interactions between the complement of hydrophobic residues at the docking interface [20]. Second, it is clear that multiple protein*–*protein interactions, other than those between the docking domains, can influence the efficiency and specificity of polyketide transfer. For example, truncated PKS proteins lacking N-terminal docking domains can nevertheless catalyze polyketide chain extension [25], and interprotein interactions between the C-terminal ACP domain and N-terminal KS domain of adjacent proteins can mitigate the effect of mismatched docking domains [27]. If such interactions are relevant, it is likely that they will influence residue co-evolution, both between docking domains as well as between other domain varieties. At our current level of sensitivity, we were able to identify a handful of residue pairs contributing to the specificity of *H1–T1* interactions, but were unable to find any significant pairs for the other compatibility classes. Moreover, when we applied the same analysis to the case of interactions between the much larger ACP and KS domains, we found no significant co-evolution (Figures S1E and S1F). If we are to increase the sensitivity of our analysis, we must increase the number of ordered PKS pathways in our dataset. Aside from waiting for more PKS pathways to be biochemically characterized, one way to achieve this would be to include putative pathways from fully sequenced genomes whose order was inferred using techniques such as those developed by Minowa et al. [10] (though this runs the risk of circularity).

### Hierarchy, Modularity, and Abstraction

One of our central findings is that the PKS specificity code is hierarchical. At the highest level, there are phylogenetically diverged, extremely distinct compatibility classes of docking domains; at the next level, there are subclasses of domains that essentially differ from one another at just a few residues. As such, it is possible to achieve interactions between any pair of docking domains in a given phylogenetic class by a handful of mutations, but docking domains from different phylogenetic classes are likely to remain forever incompatible. This hierarchical organization provides important clues about the selective pressures that operate on PKS pathways. If it were possible to switch the class of any docking domain to any other by mutation, undesirable interactions in PKS multiprotein chains would arise at high frequency, reducing the overall fitness of a bacterial population. At the opposite extreme, if docking domains were all extremely distinct from one another, it would be prohibitively difficult to “reprogram” the order of a PKS multiprotein chain, so bacteria would not be sufficiently nimble in response to rapidly changing ecological conditions. The observed hierarchy of classes and subclasses might represent an optimal intermediate strategy, balancing the competing requirements of robustness and flexibility.

**Figure 6:**
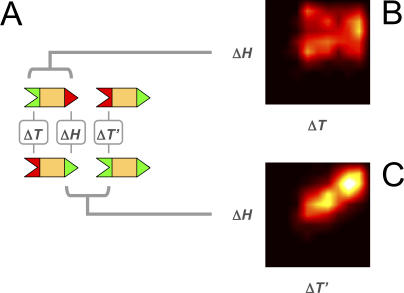
Domain Shuffling and Domain Linkage (A) Δ*H* is the fraction of amino acid differences between some pair of head domains; Δ*T* is the fraction of amino acid differences between some pair of tail domains. We compare the observed degree of similarity of two head domains (Δ*H*), with that of the two tail domains on the same proteins (Δ*T*) or the two tail domains that are their interaction partners (Δ*T′*). Each such comparison gives us a point in Δ*H −* Δ*T* space; by running over all possible pairs, we generate a family of points. (B,C) Density plots of points in Δ*H −* Δ*T* space, with axes running from 0 (identical) to 1 (distinct). To generate these plots, a 2-D histogram was calculated by binning the data into a 10-by-10 grid, which was then smoothed by interpolation. The multimodal appearance of these plots is a manifestation of the underlying phylogenetic clusters. (B) Density plot of Δ*H* versus Δ*T*, when the head and tail domains belong to the same protein. *A priori*, we expect two proteins descended from a common ancestor to show a uniform degree of sequence similarity across their entire length. Instead, we find that Δ*H* is largely uncorrelated with Δ*T* (cc = 0.13). That is, proteins with very similar head domains can have very diverged tail domains, and vice versa. This implies that proteins are not inherited in their entirety, but instead undergo frequent domain shuffling. (C) Density plot of Δ*H* versus Δ*T′*, when the head and tail domains are interaction partners. In this case, Δ*H* and Δ*T′* are highly correlated (cc = 0.67), implying that these two domains are evolutionarily linked. Remarkably, the interacting domain pair straddling two proteins, rather than the protein itself, constitutes the true unit of inheritance.

If we examine how PKS pathways are encoded at the genetic level, we uncover two further puzzles. First, why are the PKS docking domains positioned so close to protein termini (Figure 1A), when it is not uncommon to find protein*–*protein interaction domains deep within protein coding regions? Second, why does gene order tend to match protein order in PKS pathways, when experiments involving hybrid PKSs [23,24] have shown that gene order is not essential for function? The key to both these puzzles lies in the patterns of PKS pathway inheritance. If proteins were inherited in their entirety over evolutionary timescales, we would expect their N- and C-termini to have similar phylogenetic trees. Instead, we find that pairs of proteins with closely related tails can have distantly related heads and vice versa (Figure 6B), implying that domain shuffling occurs frequently. More remarkably, we find that the interacting domain pairs, the head of one protein and the tail of its partner, have similar phylogenies (Figure 6C). Interacting docking domains, *straddling two proteins*, constitute the unit of inheritance. This is presumably because the two also represent a unit of function, one being useless without the other. Their combined inheritance is ensured if these two protein fragments are encoded contiguously on the genome. Indeed, we find that most of the docking domain pairs in our dataset are adjacently transcribed (Dataset S2). Of course, this would require that docking domains map to gene termini, and that gene order match protein order. In a world of rampant recombination and gene transfer, domain and gene order are functionally irrelevant, but evolutionarily important.

The high-level structure of the PKS protein interaction code is striking. The *hierarchical* organization of docking domains into compatibility classes and subclasses creates multiprotein chains that are simultaneously robust and reprogrammable. A pair of interacting docking domains form a single *module*, a genetic unit straddling protein termini, so they are always inherited together. And the correspondence between gene order and protein order is a beautiful example of *abstraction*, allowing new protein configurations to be efficiently sampled through the underlying process of DNA recombination. All this amounts to a common standard for information exchange, allowing microbes to access a shared pool of biosynthetic capabilities. It would appear that PKS pathways should not simply be regarded as machines, evolved to produce this or that polyketide product. Rather, they represent a sort of genetic sketchpad, allowing biosynthesis to be abstractly represented, shared, and shuffled, in a process of continual biochemical innovation.

## Methods

### CRoSS algorithm: Correlated residues of statistical significance.

Head and tail domains of interest were grouped by PKS pathway. For each pathway, known interactors (I) and known noninteractors (NI) were coupled, and appropriately weighted. For example, consider a hypothetical pathway which involves five proteins, with m_I_ = 4 head–tail interfaces. Let the heads be labeled h_i_ and the tails t_i_, numbered by interface i = 1, ..., m_I_. The I dataset will contain the m_I_ = 4 interacting pairs {h_1_, t_1_} through {h_4_, t_4_}, each with weight w_I_ = 1. The NI dataset will contain the m_NI_ = 12 noninteracting pairs {h_1_, t_2_}, {h_1_, t_3_}, ..., {h_4_, t_2_}, {h_4_, t_3_}, each with a weight w_NI_ = m_I_/m_NI_ = 1/3. The total contribution of this pathway to both the I and the NI dataset will therefore be:

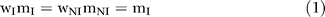



We carried out this procedure for all pathways, leaving out those with m_NI_ = 0. The datasets I and NI each contain an ordered list of head–tail pairs indexed by r, with corresponding weights w_r_. The sequences of the rth domain pair are represented by binary variables as follows: 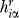 , 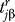 , where i runs over head sites and j over tail sites, and α and β run over the 20 amino acids. We next calculated the joint distribution of amino acids at some site pair {i, j}:

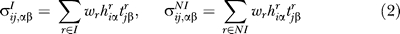



For any given site pair {i, j}, it follows from Equation 1) that the marginal distribution of amino acids are identical:

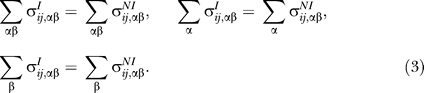



Therefore, the only way the joint distributions can be different is if they display different correlations. However, we must be careful not to take the observed correlations too seriously, given the small size of the dataset. We therefore calculated the significance of the difference between the joint distributions using a chi-squared test [28]:

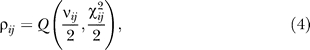
where

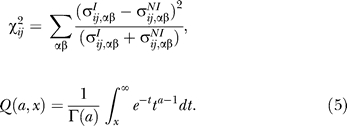



Here, ν_ij_ is the number of bins for which either 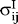 or 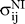 is nonzero, Q is the incomplete gamma function, and Γ(*a*) is the Euler gamma function.

If this were a true chi-squared test, the final CRoSS matrix ρ_ij_ would give the probability that the interaction and noninteraction distributions were drawn from the same underlying joint-distribution. The lower this value, the more significant the site pair {i, j} as a predictor of specificity. In fact, the fractional weighting used in Equation 1) means that the values ρ_ij_ overestimate the probability of the null hypothesis. Moreover, we are testing multiple hypotheses, one for each site pair under consideration. In choosing a threshold to classify a particular subset of site pairs as being significant, it is useful to compare the distribution of values ρ_ij_ against a background distribution ρ_ij_, generated by applying CRoSS to compare random pairings with noninteractors. This is how the seven significant pairs in Figure 3B were identified (Figure S1C).

CRoSS was implemented in MATLAB (The MathWorks).

## Supporting Information

Dataset S1 List of PKS Pathways, Abbreviations, and Data Sources (11 KB TXT)

Dataset S2 Compatibility Class Multiple Sequence Alignments (13 KB TXT)

Figure S1 Selecting Significant CRoSS Residue Pairs (A) The *H1–T1* control matrix (also shown in Figure 3A), generated by using CRoSS to compare random pairings with noninteractors, has entries -log_10_(*ρ* ^0^ *_ij_*).
(B) The *H1–T1* interaction matrix (also shown in Figure 3B), generated by using CRoSS to compare interactors with noninteractors, has entries -log_10_(*ρ_ij_*).
(C) Cumulative histograms of the values log_10_(*ρ* ^0^ *_ij_*) (blue) and log_10_(*ρ_ij_*) (red). The distribution of *ρ-*values in the CRoSS interaction matrix coincides with that of the control matrix above *ρ* ∼ 10^−1^. However, the CRoSS interaction matrix shows a tail of much lower *ρ*-values. The seven lowest points were selected as representing significant CRoSS pairs.
(D) Significance and mutual information. For each site pair, we know the joint distribution of amino acids for interactors, as well as for noninteractors (Methods, Equation 2). CRoSS calculates a significance score *ρ*, which reports the probability that these two observed distributions could arise from the same underlying distribution. In screening for co-evolving pairs, an alternative approach would be to use the interactor joint distribution alone, and to measure the mutual information between the head and tail amino acids (closely related to the approach used in [17] and [18]). To compare these two approaches, we plotted mutual information (in natural logarithms) against the significance *ρ*, for all site pairs. The two most significant points have the highest mutual information, and all seven chosen points (red) have high mutual information as expected, since they are useful predictors of specificity. However, there is a cloud of points of low significance (black) which show a spread of mutual information values. Selecting site pairs based on mutual information alone would tend to include a large number of spurious correlations, or to exclude most of the important correlations.
(E*–*F) Interactions between ACP and KS domains have been shown to contribute to specificity [27]. We analyzed the C-terminal ACP domains and N-terminal KS domains adjacent to the 90 *H1–T1* docking domain pairs in our dataset, precisely as we had done for the docking domains themselves. Starting with a multiple-sequence alignment, we applied CRoSS to detect co-evolving residues by comparing interactors with noninteractors (*ρ_ij_*). We also applied CRoSS on a control dataset, comparing random pairings with noninteractors (*ρ* ^0^ *_ij_*).
(E) The ACP-KS CRoSS interaction matrix shows very low signals (note the color scale, which is different from that used in Figure S1B).
(F) CRoSS entries in the interaction (red) and control (blue) matrices are comparable, indicating that there are no significant co-evolving pairs.
(1.6 MB PDF)

Figure S2 Measuring the Predictive Ability of the Subclass Code (A) Receiver operating characteristics (ROC) for predictions based on Monte Carlo clustering. Each curve shows the TP rate versus the FP rate, as the clustering threshold *N_min_* is varied from high (TP = FP = 0) to low (TP = FP = 1). Curves of different colors correspond to different partitions of the data into training and test sets.
B) Interpolated ROC curves were used to calculate the FP value at each TP value, for each of the 15 training and test sets. Here we show the mean ROC value (black) along with one standard error on each side (gray) as a function of TP values. A random classifier would trace the curve TP = FP (red), while the ROC of a classifier that performed better than random would lie above this diagonal. The performance of our code is marginally better than random, but statistically significant. The performance tends to be better at higher FP values. The mean area under the ROC is 0.55 ± 0.02, and at FP = 0.5, we have TP = 0.6.
(C) Performance of the code as a predictor of PKS multiprotein chain order. Consider a hypothetical five-protein chain for which the termini (1, 5) are specified, so the three internal proteins (2, 3, 4) can have six possible permutations. Using FP = 0.5 and TP = 0.6, we generated a possible prediction of pairwise docking domain interactions based on the correct underlying multiprotein chain permutation. We then used Bayesian inference to assign a posterior probability to the six possible permutations given the prediction, and selected the permutation with the maximum posterior probability. (The six possible permutations correspond to six allowed types of pairwise interactions between the four internal head*–*tail pairs of the multiprotein chain. These are represented as six 4 × 4 matrices with a single entry in each row and column.) This procedure was repeated for 1,000 trials. The figure shows the fraction of trials in which each of the six possible permutations was selected as being most likely. We see that the correct permutation is chosen in 42% of instances. Random guessing would pick the correct permutation in 16.7% of instances, so our performance is 2.5 times better than random.
(1.1 MB PDF)

Text S1 Supporting Analysis (281 KB PDF)

## Abbreviations

CRoSS: correlated residues of statistical significance
FP: false positive
PKS: polyketide synthase
ROC: receiver operating characteristics
TP: true positive
