# Supplementary figures and images for: The Origins of Specificity in Polyketide Synthase Protein Interactions
Source: PLoS Comput Biol. 2007 Sep 28;3(9):e186. doi: 10.1371/journal.pcbi.0030186 (PMC1994986; doi:10.1371/journal.pcbi.0030186)

A

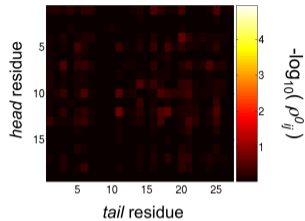

B

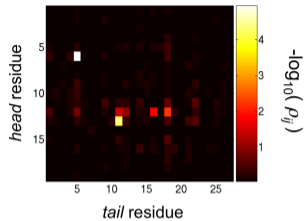

C

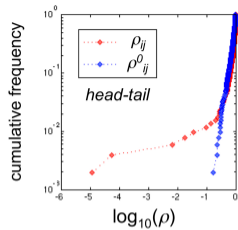

D

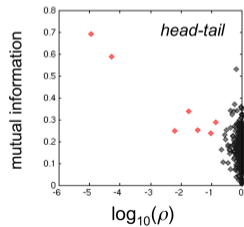

E

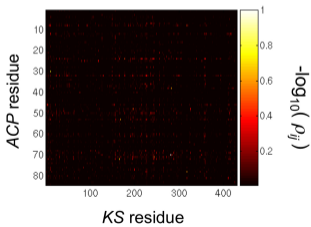

F

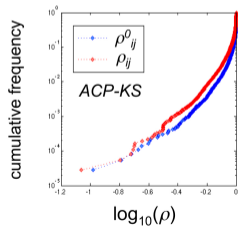

Supplement: Figure S1 — (A) The H1–T1 control matrix (also shown in Figure 3A), generated by using CRoSS to compare random pairings with noninteractors, has entries -log10(ρ 0 ij). (B) The H1–T1 interaction matrix (also shown in Figure 3B), generated by using CRoSS to compare interactors with noninteractors, has entries -log10(ρij). (C) Cumulative histograms of the values log10(ρ 0 ij) (blue) and log10(ρij) (red). The distribution of ρ-values in the CRoSS interaction matrix coincides with that of the control matrix above ρ ∼ 10−1. However, the CRoSS interaction matrix shows a tail of much lower ρ-values. The seven lowest points were selected as representing significant CRoSS pairs. (D) Significance and mutual information. For each site pair, we know the joint distribution of amino acids for interactors, as well as for noninteractors (Methods, Equation 2). CRoSS calculates a significance score ρ, which reports the probability that these two observed distributions could arise from the same underlying distribution. In screening for co-evolving pairs, an alternative approach would be to use the interactor joint distribution alone, and to measure the mutual information between the head and tail amino acids (closely related to the approach used in [17] and [18]). To compare these two approaches, we plotted mutual information (in natural logarithms) against the significance ρ, for all site pairs. The two most significant points have the highest mutual information, and all seven chosen points (red) have high mutual information as expected, since they are useful predictors of specificity. However, there is a cloud of points of low significance (black) which show a spread of mutual information values. Selecting site pairs based on mutual information alone would tend to include a large number of spurious correlations, or to exclude most of the important correlations. (E–F) Interactions between ACP and KS domains have been shown to contribute to specificity [27]. We analyzed the C-terminal ACP domains [file pcbi.0030186.sg001.pdf]

A

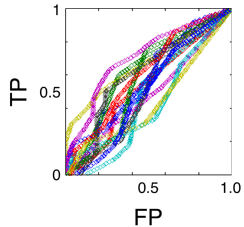

B

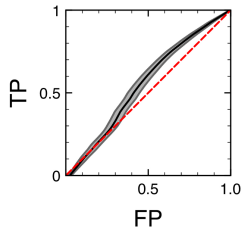

C

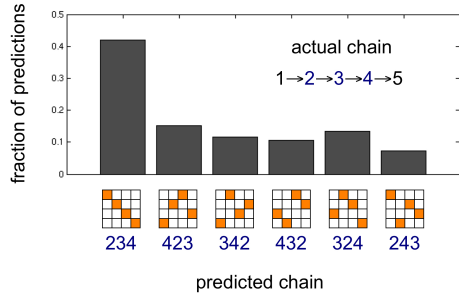

Supplement: Figure S2 — (A) Receiver operating characteristics (ROC) for predictions based on Monte Carlo clustering. Each curve shows the TP rate versus the FP rate, as the clustering threshold Nmin is varied from high (TP = FP = 0) to low (TP = FP = 1). Curves of different colors correspond to different partitions of the data into training and test sets. B) Interpolated ROC curves were used to calculate the FP value at each TP value, for each of the 15 training and test sets. Here we show the mean ROC value (black) along with one standard error on each side (gray) as a function of TP values. A random classifier would trace the curve TP = FP (red), while the ROC of a classifier that performed better than random would lie above this diagonal. The performance of our code is marginally better than random, but statistically significant. The performance tends to be better at higher FP values. The mean area under the ROC is 0.55 ± 0.02, and at FP = 0.5, we have TP = 0.6. (C) Performance of the code as a predictor of PKS multiprotein chain order. Consider a hypothetical five-protein chain for which the termini (1, 5) are specified, so the three internal proteins (2, 3, 4) can have six possible permutations. Using FP = 0.5 and TP = 0.6, we generated a possible prediction of pairwise docking domain interactions based on the correct underlying multiprotein chain permutation. We then used Bayesian inference to assign a posterior probability to the six possible permutations given the prediction, and selected the permutation with the maximum posterior probability. (The six possible permutations correspond to six allowed types of pairwise interactions between the four internal head–tail pairs of the multiprotein chain. These are represented as six 4 × 4 matrices with a single entry in each row and column.) This procedure was repeated for 1,000 trials. The figure shows the fraction of trials in which each of the six possible permutations was selected as being most likely. We see that the correct permuta [file pcbi.0030186.sg002.pdf]
