# Supplementary material for: The Origins of Specificity in Polyketide Synthase Protein Interactions
Source: PLoS Comput Biol. 2007 Sep 28;3(9):e186. doi: 10.1371/journal.pcbi.0030186 (PMC1994986; doi:10.1371/journal.pcbi.0030186)
Supplement: Text S1 — (281 KB PDF) [file pcbi.0030186.sd003.pdf]

The Origins of Specificity in Polyketide Synthase Protein Interactions

Mukund Thattai[1], Yoram Burak[2], Boris I. Shraiman[2]

1: National Centre for Biological Sciences, UAS Campus, Bellary Road, Bangalore 560065, India.
2: Kavli Institute for Theoretical Physics, Kohn Hall, UCSB, Santa Barbara, CA 93106, USA.

Text S1: Supporting Analysis

Contents:

1. Assembly of the dataset 2
2. Compatibility classes: clustering of head and tail docking domains........ 2
3. Compatibility sub-classes: Monte Carlo clustering of code words........ 2
4. Mapping CRoSS residues to the docked domain NMR structure........ 4
5. Measuring the predictive ability of the specificity code........ 5
References........ 7

1. Assembly of the dataset. Seed PKS protein sequences were obtained from the PKSDB database [7]. Conserved N- and C-terminal regions defined from a preliminary alignment were fed separately through PSI-BLAST [S1], to find matches in the non-redundant protein database. The PSI-BLAST cutoff was chosen so as to include all proteins annotated as "synthases", and subsequent iterations were performed until convergence. NCBI annotations of the resulting proteins were scanned manually, to determine primary literature references. All papers with two or more protein hits were included. The resulting 42 papers all reported studies of modular PKSs (Dataset S1, Section 1). All 225 PKS proteins obtained from these references were combined to form our raw dataset.

200 aa of the C-terminal and 50 aa of the N-terminal regions of these proteins were aligned using MUSCLE multiple sequence alignment software [S2]. The alignment was run for 10 iterations, with a gap-opening penalty of -10. The dataset was then pruned in two steps. All obvious badly aligned proteins were removed. Of the result, all termini without interaction partners were removed. These included the pathway termini (the N-terminal region of the first protein and the C-terminal region of the last protein in a PKS pathway), which typically tended not to align. The resulting sequences were then re-aligned using the same parameters. The alignments showed conserved regions at the very ends of proteins, followed by gaps or unconserved linkers: a 19 aa C-terminal "head" region, and a 27 aa N-terminal "tail" region. The final dataset consisted of 149 head-tail interaction partners. (The alignments shown in Figure 1 were rendered using Jalview [29].)

2. Compatibility classes: clustering of head and tail docking domains. The CLANS tool [16] can be used to rapidly investigate phylogenetic relationships in large datasets. CLANS was used to perform a force-based clustering of head and tail domains, as defined in Section 1, using BLAST cutoffs of 2.0e-4 and 1.0e-4, respectively. Stringent cutoffs were chosen so as to resolve separate clusters; more lenient cutoffs tend to group all homologous domains into a single large cluster. The system was run in 2-dimensions; the attract and repel coefficients were set to 10, and the corresponding exponents were set to 2. The system was allowed to evolve till equilibrium. Heads and tails were assigned to clusters by hand, based on the CLANS output in 2-dimensions. The clusters reported here largely reproduce those predicted by other phylogenetic reconstruction algorithms.

3. Compatibility sub-classes: Monte Carlo clustering of code words. We have two distinct goals from clustering. The first is to identify groups of amino acid code words that obey similar interaction rules; the second is to determine the "clusterability" of the data compared to that of a random dataset. We say that a dataset is "clusterable" when: (i) code words group into cliques, within which pairs are likely to be interactors, and between which pairs are likely to be non-interactors; and (ii) cliques contain words of similar sequence.

We begin by including all CRoSS pairs with significance more than some cutoff, thus restricting our attention to a subset of sites on the head and tail domains. For any given domain instance, the residues at these sites define short amino-acid code words; the same code word typically occurs in multiple domain instances. The code words are represented by binary variables $h^r_{i\alpha}$, $t^s_{j\beta}$, where i runs over head sites and j over tail sites, and α and β run over the 20 amino acids. The interaction data can be represented on a bi-partite graph (Figure 4B): the nodes are unique head or tail code words, indexed by r and s, respectively; the edges e_{rs} connect head nodes to tail nodes, representing all unique interactions (e_{rs} = +1) or non-interactions (e_{rs} = -1). In addition, the nodes are each labeled with an integer λ_r or λ_s, such that nodes with the same label belong to the same cluster.

We next calculate two energy terms. The first term, an interaction energy, is defined as:

$$\begin{aligned} H_{int} = &-\sum_{e_{rs}=+1} q\delta(\lambda_r,\lambda_s) + w(1-q)(1-\delta(\lambda_r,\lambda_s)) \\ &-\sum_{e_{rs}=-1}(1-q)\delta(\lambda_r,\lambda_s) + wq(1-\delta(\lambda_r,\lambda_s)) \end{aligned} \qquad [S1]$$

Here, $\delta(a,b) = 1$ if $a = b$, and is 0 otherwise; $0 \le q \le 1$ sets the relative contribution of desirable versus undesirable edges; and $w > 0$ sets the relative contribution of between-cluster to within-cluster terms. For example, with q = 0.8 and w = 1.0, interaction edges (e_{rs} = +1) contribute an energy 0.8 if they occur within clusters, but 0.2 if they occur between clusters; non-interaction edges (e_{rs} = -1) contribute 0.2 within clusters, but 0.8 between clusters. This term thus tends to favor interactions within clusters, and non-interactions between clusters.

The second term, an entropic contribution, is defined as a sum over clusters:

$$H_{ent} = -\sum_{\lambda}\left[\sum_r \delta(\lambda,\lambda_r)\sum_{i\alpha} h^r_{i\alpha}\ln(p^\lambda_{i\alpha}) + \sum_s \delta(\lambda,\lambda_s)\sum_{j\beta} t^s_{j\beta}\ln(p^\lambda_{j\beta})\right], \qquad [S2]$$

where cluster-specific amino-acid frequencies, normalized at each site, are given by

$$p^\lambda_{i\alpha} = \frac{\sum_r \delta(\lambda,\lambda_r) h^r_{i\alpha}}{\sum_{\alpha'}\sum_r \delta(\lambda,\lambda_r) h^r_{i\alpha'}}, \qquad p^\lambda_{j\beta} = \frac{\sum_s \delta(\lambda,\lambda_s) t^s_{j\beta}}{\sum_{\beta'}\sum_s \delta(\lambda,\lambda_s) t^s_{j\beta'}} \qquad [S3]$$

Essentially, we are measuring the log-odds that any given head or tail code word will appear in a cluster, given the average amino-acid frequencies for that cluster. This term tends to reward clusters which contain code words that are similar to one another in sequence.

For any given partitioning of the nodes into labeled clusters, the interaction and entropic terms are finally weighted and combined to give a total energy

$$H = w_{int} H_{int} + w_{ent} H_{ent} . \qquad [S4]$$

We can now perform a Metropolis Monte Carlo simulation [22,S3] to cluster heads and tails. The system is initialized such that all head and tail nodes belong to distinct clusters; at each timestep, a random node is picked and added to an existing cluster, or is used to seed a fresh cluster. This move, which changes the energy from H to H', is accepted if $\exp(H - H') > \rho$, where ρ is a random number uniformly distributed between 0 and 1. This simulation is repeated for N_{trial} trials, always starting with a non-clustered initial condition, and run till approximate equilibrium. Each such run generates a possible partitioning of the nodes into labeled clusters.

To generate the data shown in Figure 4, we performed Monte Carlo clustering using the three most significant CRoSS site pairs, which picks out three head sites and three tail sites. We used the following parameters: $q = 0.8$; $w = 1.0$; $w_{int} = 4.0$; $w_{ent} = 0.5$; $N_{trials} = 100$. If two nodes were observed to be in the same cluster in more than N_{min} trials, they were defined as being co-clustered. The final clusters shown in Figure 4 are robust, remaining essentially unchanged as N_{min} is varied from 10 to 35; the cluster *H1b-T1b* breaks into two pieces at $N_{min} \sim 30$.

In order to estimate the significance of the clusters detected above, we ran the same algorithm on randomized datasets. These datasets were generated by swapping edges from the true dataset at random, such that the number of positive and negative edges connected to each node remained unchanged. For each randomized dataset, the simulation was run for 10 trials to estimate mean equilibrium energies; this procedure was repeated for 50 such datasets. The results were as follows:

True dataset:	$\langle H \rangle = -169.4$,	$\langle H_{int} \rangle = -51.7$,	$\langle H_{ent} \rangle = 75.0$.
Randomized datasets:	$\langle H \rangle = -134.7 \pm 9.3$,	$\langle H_{int} \rangle = -45.2 \pm 2.6$,	$\langle H_{ent} \rangle = 92.2 \pm 7.3$.

The values of both interaction and entropic energies are significantly lower for the true dataset than for the randomized datasets, indicating that interactions are more enriched within cliques, and code words are more similar, than expected by chance. Based on these 50 trials, we can conservatively set an upper bound, p-value < 0.02.

4. Mapping CRoSS residues to the docked domain NMR structure. An NMR structure for the docked complex between the C-terminus of protein 2 and N-terminus of protein 3 of the erythromycin PKS has been published by Broadhurst *et al.* [15], with PDB code 1pzr. We used the CSU contact analysis tool [S4] to determine pairwise distances between head and tail residues in the NMR structure. Since PKS proteins exist as homodimers, there are two copies each of any given head or tail residue, and therefore four distances associated with any given residue pair. Of

these, we selected the minimum pairwise distance. Figure 5A shows the residue pairs that are separated by a distance of 5Å or less in the NMR structure. On the same figure, we have highlighted the residue pairs selected by CRoSS, as well as those previously suggested or demonstrated as contributing to interaction specificity. Broadhurst *et al.* used an unstructured linker to connect the C- and N-termini, which were subsequently permitted to dimerize and dock with one-another. In Figures 5B and 5C, we omit the linker region for clarity. Figure 5B was generated using BALLVIEW [S5], and Figures 5C-5F were generated using CN3D [S6].

5. Measuring the predictive ability of the specificity code. In order to rigorously test the predictive power of our code, we must train it on one dataset, and test it against an independent dataset. This allows us to estimate how well the code is able to generalize to previously unseen data, in terms of true positive and false positive rates. We carried out this procedure separately for each level of the code.

Compatibility classes: In the original analysis, we used the CLANS tool to cluster head and tail docking domains into compatibility classes (Text S1, Section 2). However, CLANS is a clustering algorithm which simultaneously manipulates a set of sequences, rather than classifying individual ones. As such, it cannot be trained on one dataset and tested on another. In order to measure the ability of our code to generalize, we adopted the following approach. We took the original CLANS classification as given, and asked whether, knowing the classification of one subset of domains, we could predict that of another. Our complete dataset consists of 128 matched head-tail pairs (the diagonal interactors in Figure 2C), classified as *H1-T1*, *H2-T2*, or *H3-T3*. We split these at random into 96 training cases, and 32 test cases. Based on the examples in the training set, we built six position-specific weight matrices (PSWMs, giving the probability of finding a given amino acid at each site) for each of the six domain varieties *H1*, *H2*, *H3* and *T1*, *T2*, *T3*. Next, we applied these PSWMs to classify all the domains in our test set, based on maximum likelihood. (For example, given any head domain, we calculated the probability that it could arise from each of the three PSWMs corresponding to *H1*, *H2*, and *H3*, and assigned it to the class that produced the highest probability.) *A priori*, we would predict that a matched head-tail pair would interact, while a mis-matched head-tail pair would not. Now, of the 32x32 possible head-tail pairings within the test dataset, we will have 32 known interactors (the diagonal elements) as well as some number of known non-interactors (non-interacting pairs from the same PKS pathway). The true positive (TP) rate is the fraction of known interacting pairs that are matched, while the false positive (FP) rate is the fraction of known non-interacting pairs that are matched. By repeating this procedure for 1000 trials of randomly divided training and test sets, we found that the compatibility class code performs with TP = 0.97 ± 0.03, FP = 0.52 ± 0.2.

Compatibility sub-classes: If we use the 3 most significant CRoSS residue pairs, our code word graph contains 34 head code words and 27 tail code words as nodes, with 55 interaction edges and 130 non-interaction edges connecting them. We can measure the performance of the sub-class specificity code by asking how often the nature of a new edge can be predicted. To estimate

this, we split our edge dataset at random into 150 training edges, and 35 test edges, and ran the Monte Carlo clustering algorithm described above for 50 trials on the training set. A particular choice of the threshold N_{min} induces a particular clustering on the nodes, allowing us to ask if the edges in the test set connect nodes within a cluster (predicted interactors), or between clusters (predicted non-interactors). If we define the values TP (true positives: the fraction of interactor edges in the test set that are predicted to be interactors) and FP (false positives: the fraction of non-interactor edges in the test set that are predicted to be interactors), then high N_{min} gives TP = FP = 0, while low N_{min} gives TP = FP = 1. Intermediate values of N_{min} trace out a curve of TP vs. FP, known as the Receiver Operating Characteristic (ROC) [S7]. A random classifier would trace the curve TP = FP, while the ROC of a classifier that performed better than random would lie above this diagonal. A typical measure of performance is the area under the ROC curve, which should be greater than 0.5 for a better-than-random classifier. We calculated ROCs for 15 different randomly split training and test datasets (Figures S2A and S2B). The classifier performs slightly better than random, with the improvement being greater at higher FP rates. The mean area under the ROC is 0.55 ± 0.02, and at FP = 0.5, we have TP = 0.6. This poor performance is due to the sparseness of the available dataset, in which removing even a few edges disrupts certain clusters. (If we use the 4 most significant CRoSS residue pairs, we find that the mean area under the ROC is 0.5 -- the code is unable to generalize, performing no better than random guessing on test data.) However, even at this level of performance the code can be usefully applied. For example, a typical application would be to infer the correct order of a novel PKS multi-protein chain, given the sequences of its docking domains. Consider a hypothetical five-protein chain for which the termini are specified, so the three internal proteins can have six possible permutations. If the code performs at FP = 0.5 and TP = 0.6, we are able to predict the correct permutation in 42% of cases, or 2.5 times the random rate (Figure S2C).

References

S1. Altschul SF, Madden TL, Scher AA, Zhang J, Zhang Z, Miller W, Lipman DJ (1997) Gapped BLAST and PSI-BLAST: a new generation of protein database search programs. Nucleic Acids Res. 25: 3389-3402.

S2. Edgar RC (2004) MUSCLE: a multiple sequence alignment method with reduced time and space complexity. *BMC Bioinformatics* 5: 113-131.

S3. Ma S-K (1985) Statistical Mechanics. Philadelphia PA: World Scientific. pp. 391-397.

S4. Sobolev V, Sorokine A, Prilusky J, Abola EE, Edelman M (1999) Automated analysis of interatomic contacts in proteins. Bioinformatics 15: 327-332.

S5. Moll A, Hildebrandt A, Lenhof HP, Kohlbacher O (2006) BALLView: a tool for research and education in molecular modeling. *Bioinformatics* 22: 365-366.

S6. Wang Y, Geer LY, Chappey C, Kans JA, Bryant SH (2000) Cn3D: sequence and structure views for Entrez. *Trends Biochem Sci*. 25: 300-302.

S7. Egan JP (1975) Signal detection theory and ROC analysis. New York NY: Academic Press.
